# Effect of divalent Ba cation substitution with Sr on coupled ‘multiglass’ state in the magnetoelectric multiferroic compound Ba_3_NbFe_3_Si_2_O_14_

**DOI:** 10.1038/srep09751

**Published:** 2015-05-19

**Authors:** Satyapal Singh Rathore, Satish Vitta

**Affiliations:** 1 Indian Institute of Technology Bombay, Mumbai 400076, India

## Abstract

(Ba/Sr)_3_NbFe_3_Si_2_O_14_ is a magneto-electric multiferroic with an incommensurate antiferromagnetic spiral magnetic structure which induces electric polarization at 26 K. Structural studies show that both the compounds have similar crystal structure down to 6 K. They exhibit a transition, T_N_ at 26 K and 25 K respectively, as indicated by heat capacity and magnetization, into an antiferromagnetic state. Although Ba and Sr are isovalent, they exhibit very different static and dynamic magnetic behaviors. The Ba-compound exhibits a glassy behavior with critical slowing dynamics with a freezing temperature of ~35 K and a critical exponent of 3.9, a value close to the 3-D Ising model above T_N_, in addition to the invariant transition into an antiferromagnetic state. The Sr-compound however does not exhibit any dispersive behavior except for the invariant transition at T_N_. The dielectric constant reflects magnetic behavior of the two compounds: the Ba-compound has two distinct dispersive peaks while the Sr-compound has a single dispersive peak. Thus the compounds exhibit coupled ‘multiglass’ behavior. The difference in magnetic properties between the two compounds is found to be due to modifications to super exchange path angle and length as well as anti-site defects which stabilize either ferromagnetic or antiferromagnetic interactions.

The search for materials wherein the magnetization M and electric polarization P are strongly coupled has intensified recently with the discovery of magneto-electric coupling in a variety of materials^1^,^2^,^3^,^4^,^5^. In these materials both space inversion and time reversal symmetry need to be broken to realize coupling between the two orders. The linear magnetoelectric coupling constant *α_ij_*, given by 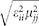 depends on the magnetic permeability tensor *μ_jj_* and electric permittivity tensor *ε_ii_* and requires that they be as large as possible for ‘strong coupling’^6^. However, most materials have either small *μ*’*s* or small *ε*’*s* or both and hence the coupling constant *α_ij_* becomes even smaller. Hence in the search for materials with strong coupling, materials with non-conventional magnetic structures such as spirals and toroids have been investigated and they have been found to exhibit strong coupling^7^,^8^,^9^,^10^,^11^. An alternative approach to overcome the limitations of linear coupling would be to investigate materials wherein higher order quadratic coupling constants become dominant compared to the linear coupling constant. This can be seen from the total free energy F expression of a homogenous, stress-free material given by;
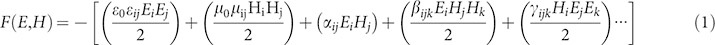
where *ε*_0_ and *μ*_0_ refer to free space permittivity and permeability respectively, *H* and *E* are magnetic and electric fields. An advantage of invoking the quadratic terms, β_ijk_ and γ_ijk_ is that the system need not fulfill the symmetry criteria which are essential for the linear coupling constant, α_ij_. Disordered multiferroics which do not fulfill the stringent symmetry criteria of linear magnetoelectric coupling belong to this class of materials and have received considerable attention in recent years. Materials with complex magnetic structure with multiple degenerate ground states are another class of materials which can have large higher order quadratic coupling constants^12^,^13^,^14^,^15^,^16^.

Recently, Ba_3_NbFe_3_Si_2_O_14_, a langasite family compound, has been shown to exhibit a frustrated, spirally ordered antiferromagnetic (AF) structure which leads to ferroelectric (FE) ordering at 26 K. Unlike classical multiferroic materials which are largely based on perovskite family compounds, this compound belongs to the langasite family of silicates. This compound has been known to have a magnetic and geometrically frustrated structure which results in initiating ferroelectric order. The magnetic Fe^3+^ (S = 5/2) ions have a triangular coplanar arrangement in the a-b plane and form a trimer with a frustrated spin structure, Figure 1. The equilateral triangular structure of Fe^3+^ ions has been found to get deformed for T < T_N_ leading to the formation of a lower symmetry polar C2 or P3 structures compared to the non-polar P321 structure present above T_N_. This trimer structure has a helical arrangement along c-axis and orders antiferromagnetically below 26 K, the Neel temperature, T_N_. The magnetic spiral is known to be commensurate with helical period of 3.66 nm, 7 times the lattice parameter along c-direction leading to an angular rotation of 51° of the magnetic moments between the different a-b planes. The helical magnetic structure is stabilized by the two in-plane (J_1_, J_2_) and three out-of-plane (J_3_, J_4_, J_5_) Fe-O-O-Fe super-exchange interactions. This magnetic structure has been found to trigger formation of a polar lattice for T < T_N_ and hence ferroelectricity^17^,^18^. The macroscopic properties of this compound however have been found to be far more complex compared to the simple structural perspective mentioned above. The magnetic contribution to the specific heat C_P _was found to rise significantly above the background value at temperatures as high as 100 K, 4 T_N_, indicating that ~ 40% of the magnetic transition entropy is released at T ≫ T_N_ and that the magnetic ordering begins far above T_N_^19^. The magnetic structure investigated using neutrons clearly showed the presence of short range magnetic order up to ~ 100 K and that a truly long range AF structure developed only for T ≤ 10 K^20^. The recent ESR studies indicate that the low T long range left handed magnetic spiral structure is established by the anisotropic Dzyaloshinskii-Moriya (DM) interactions between the Fe^3+^ moments with interaction energies of ~ 120 mK and 45 mK perpendicular and parallel to the c-axis respectively^21^,^22^. Absorption studies performed using linearly polarized THz synchrotron radiation further indicate the presence of magnons at low T and phonons dressed with currents at T ≫ T_N_. These phonons exhibit a magnetic behavior which is not due to atomic spins and a helicoidal polarization state which is incommensurate with the crystal lattice is proposed to exist even at T > T_N_^23^. This has been confirmed by recent polarization studies which show the presence of intrinsic electric polarization in the compound. The intrinsic polarization present in the absence of a magnetic field has been found to reverse its direction with increasing magnetic field^24^.

The above discussed studies clearly show that Ba_3_NbFe_3_Si_2_O_14_ with a frustrated helical antiferromagnetic ground state exhibits non-trivial magnetic orders at different temperatures and also shows a tendency for electronic phase separation. There is however a clear lack of investigations of macroscopic behavior in detail. Hence in the present work, the magnetic and electric polarizations have been studied in detail using both static and dynamic techniques. These are corroborated with investigation of the magnetic structure down to 6 K using neutrons. Since the polycrystalline form of Ba_3_NbFe_3_Si_2_O_14_ has been investigated, the effect of various structural defects including that of antisite defects is revealed in these studies. The spiral magnetic structure is a result of both in-plane and out-of-plane Fe^3+^ ionic interactions which depends strongly on the spatial coordinates of various cations and O^2−^. The divalent cation, Ba^2+^ which plays a crucial role in out-of-plane magnetic interactions has been substituted with a smaller Sr^2+^ cation to study its effect on the magnetic structure and hence macroscopic properties. It is found that substitution changes the super exchange path length and more importantly the magnetic and dielectric properties both below and above the transition temperature.

## Experimental details

For synthesizing (Ba/Sr)_3_NbFe_3_Si_2_O_14_ (BNFSO/SNFSO) compounds, solid state reaction technique was employed with high purity BaCO_3_, Nb_2_O_5_, Fe_2_O_3_, SiO_2_ and SrCO_3_ as starting materials. The powders were mixed in a ball mill and then calcined several times at 1100°C to get a homogenous single phase. This single-phase powder was then pressed into a pellet and sintered at 1150°C to get a pellet with >95% density. These sintered pellets were used for all structural and properties characterizations. The structure and chemical composition were determined using a combination of X-ray and neutron diffraction, electron microscopy and x-ray photoelectron spectroscopy (XPS). X-ray diffraction was performed using Cu-K_α_ radiation of wavelength 0.154 nm, while neutron diffraction was performed with 0.12443 nm wavelength neutrons as a function of temperature down to 6 K at the Dhruva reactor, Bhabha Atomic Research Centre. XPS was done using Mg Kα X-rays and the spectra were analysed using the standard database [NIST-XPS Database, Version 4.1 (2012); http://srdata.nist.gov/XPS]. The specific heat capacity, C_p_ was measured both as a function of temperature T and magnetic field H up to 5 T in a steady state relaxation mode using physical property measurement system. The magnetization M and susceptibility χ were measured in the temperature range 5 K to 300 K, fields up to ±3 T and frequency range 10^1^ to 10^4^ Hz using SQUID based magnetic property measurement system and vibrating sample magnetometer. The ac susceptibility was measured using 2.5 Oe excitation field without any dc field bias. The variation of dielectric constant, ε* as a function of varying temperature, 5 K to 300 K and frequency, 10^1^ to 10^6^ Hz in the presence of 0.5 V potential was studied using Novocontrol frequency–response analyser.

## Results

### X-ray and Neutron Diffraction

**Figure 2:**
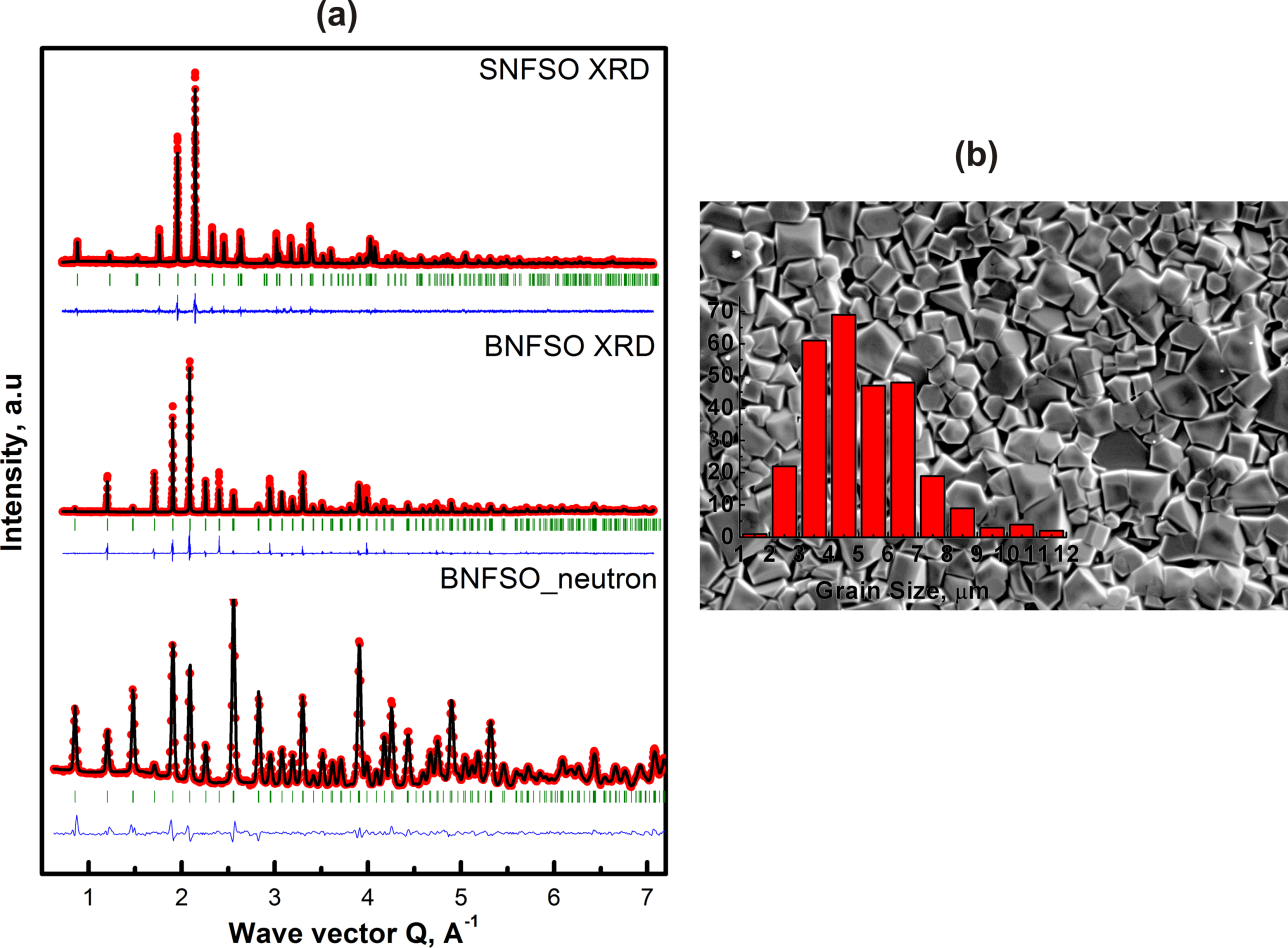
The room temperature X-ray diffraction patterns for BNFSO and SNFSO together with the neutron diffraction pattern for BNFSO are shown in (a). The structural refinement was performed using Rietveld refinement and the refined structure diffraction data is also shown together with the experimental data points. The microstructure revealed after thermal etching at 900°C clearly shows large grains as seen in the scanning electron micrograph (b). The average grain size is ~5 μm with little variation in the size.

**Figure 3:**
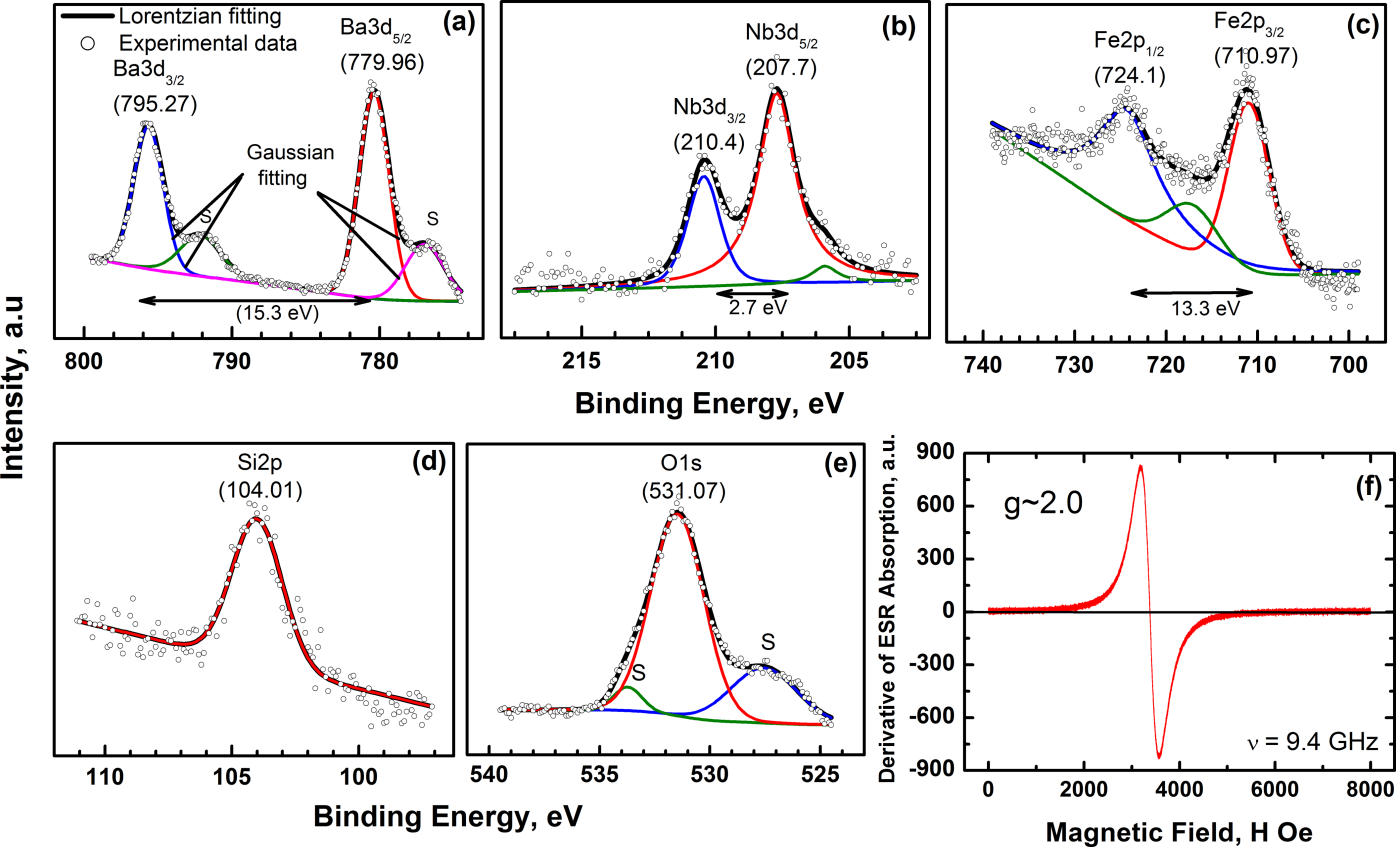
The core level XPS spectra for constituent elements in Ba_3_NbFe_3_Si_2_O_14_: Ba *3d* (a), Nb *3d* (b), Fe *2p* (c), Si *2p*(d), and O *1s*(e); the symbols represent the experimental data and solid line shows fitting. The satellite peaks are marked as S. The XPS spectra confirm that all the elements are in their expected oxidation states. (f) The electron spin resonance spectrum at room temperature also confirms the magnetic ion Fe^3+^ to be in the spin only configuration with S = 5/2.

**Figure 4:**
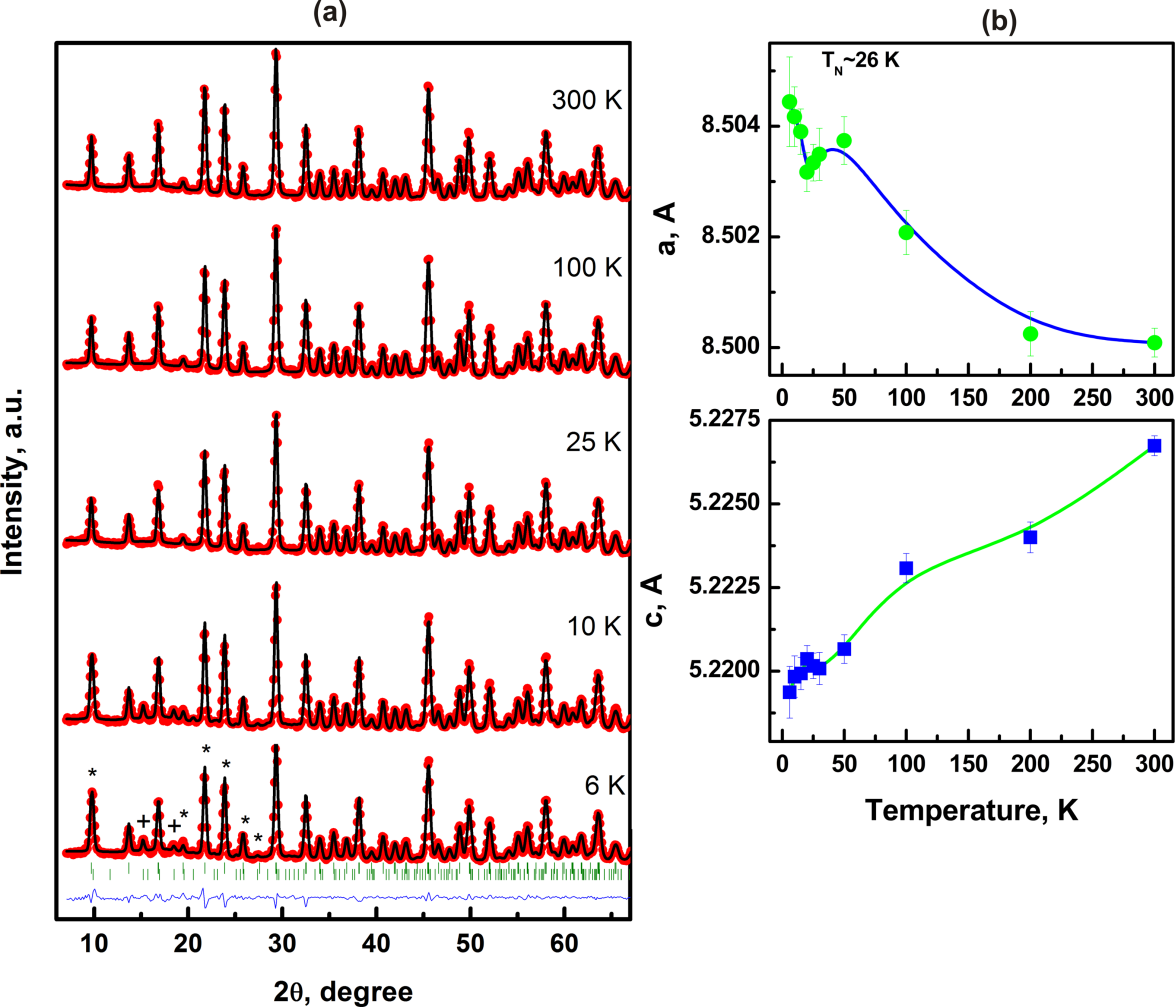
The powder neutron diffractograms obtained from Ba_3_NbFe_3_Si_2_O_4_ at different temperatures, below and above T_N_. The structural refinement clearly shows the presence of a single phase at all T and the refined data together with the experimental data is shown in (a). The unique incommensurate magnetic structure peaks are marked with * and the peaks that are common to the magnetic and crystal structure are marked with + in the 6 K diffractogram. The variation of the two lattice parameters ‘a’ and ‘c’ with T, shown in (b), has a clear peak at the transition temperature.

The room temperature X-ray diffraction patterns obtained from Ba_3_NbFe_3_Si_2_O_14_ (BNFSO) and Sr_3_NbFe_3_Si_2_O_14_ (SNFSO) are shown in Figure 2(a) along with the corresponding neutron diffraction pattern. All the reflections in both the compounds could be identified and indexed to the non-centrosymmetric non-polar hexagonal structure P321 indicating absence of any other phase. These results are in complete agreement with earlier studies performed with single crystals^17^. Since the ionic size of Sr^2+^ is less than that of Ba^2+^, this could result in variation of lattice parameters. In order to determine the changes in structural parameters, Rietveld refinement of the structure was performed and the data is fitted to the experimental pattern. The results are shown in Figure 2(a) and the crystallographic data obtained after refinement are given in Table 1. It is seen that both the lattice parameters, ‘a’ and ‘c’ indeed change and were found to decrease due to substitution of Ba^2+^ with Sr^2+^. The in-plane lattice parameter ‘a’ decreases from 0.85001 nm to 0.82605 nm while the out-of-plane lattice parameter ‘c’ decreases from 0.52267 nm to 0.51298 nm. The microstructure as seen in a scanning electron microscope, Figure 2(b) shows the presence of large grains with average grain size of 5 μm and no secondary phases. The chemical composition as well as the ionic state of all the different ions present in the compound was determined by XPS and the results are shown in Figure 3. All the peaks in the survey spectrum could be identified to the five elements Ba, Nb, Fe, Si and O indicating the purity of the compound. The core level spectra show that all the ions are in the expected ionic state, i.e. Ba^2+^, Sr^2+^, Nb^5+^, Fe^3+^, Si^4+^ and O^2−^. The presence of Fe in only the 3+ state clearly confirms the high spin, S = 5/2 state of the compounds which is also seen in the room temperature X-band electron spin resonance spectrum, Figure 3 (g). A single resonance peak is seen at a magnetic field of 3394 Oe. The gyromagnetic ratio ‘g’ determined using the resonance field is found to be 2.0 corresponding to the spin only magnetic behaviour of the compound. In order to check for structural stability/changes that can occur with changing T, temperature dependent elastic neutrons diffraction studies of Ba_3_NbFe_3_Si_2_O_14_ were undertaken and the results are shown in Figure 4(a). All the peaks in the diffraction pattern at all temperatures across the phase transition could be identified to the trigonal structure and the lattice parameters determined are shown in Figure 4(b). The two lattice parameters ‘a’ and ‘c’ vary continuously with ‘a’ decreasing while ‘c’ increasing with temperature T. The two lattice parameters however show a discontinuous change at T_N_ with a peak indicating a change in structural symmetry to a lower ordered structure^25^. Additional peaks corresponding to the magnetic order could be detected only below T_N_ indicating that the long range magnetic structure develops only for T < T_N_ with short range magnetic structural entities being present above T_N_. The presence of short range magnetic correlations in BNFSO up to T as high as 100 K have only been observed in diffuse scattering of neutrons studies^20^ and hence could not be detected in the present studies. The magnetic structure obtained by refinement of the 6 K diffraction data shows an incommensurate ordering of Fe^3+^ moments along the c-direction with the propagation vector **κ**(0,0,0.1441) corresponding to helical ordering of the magnetic moments with a period of ~7c. The effective magnetic moment is found to be ~4.2μ_B_ per Fe^3+^ ion, in agreement with earlier studies^17^,^25^. The lower value of magnetic moment is due to the partial covalent nature of Fe^3+^ electrons in the tetrahedral configuration of O^2−^ ions.

### Heat Capacity

**Figure 5:**
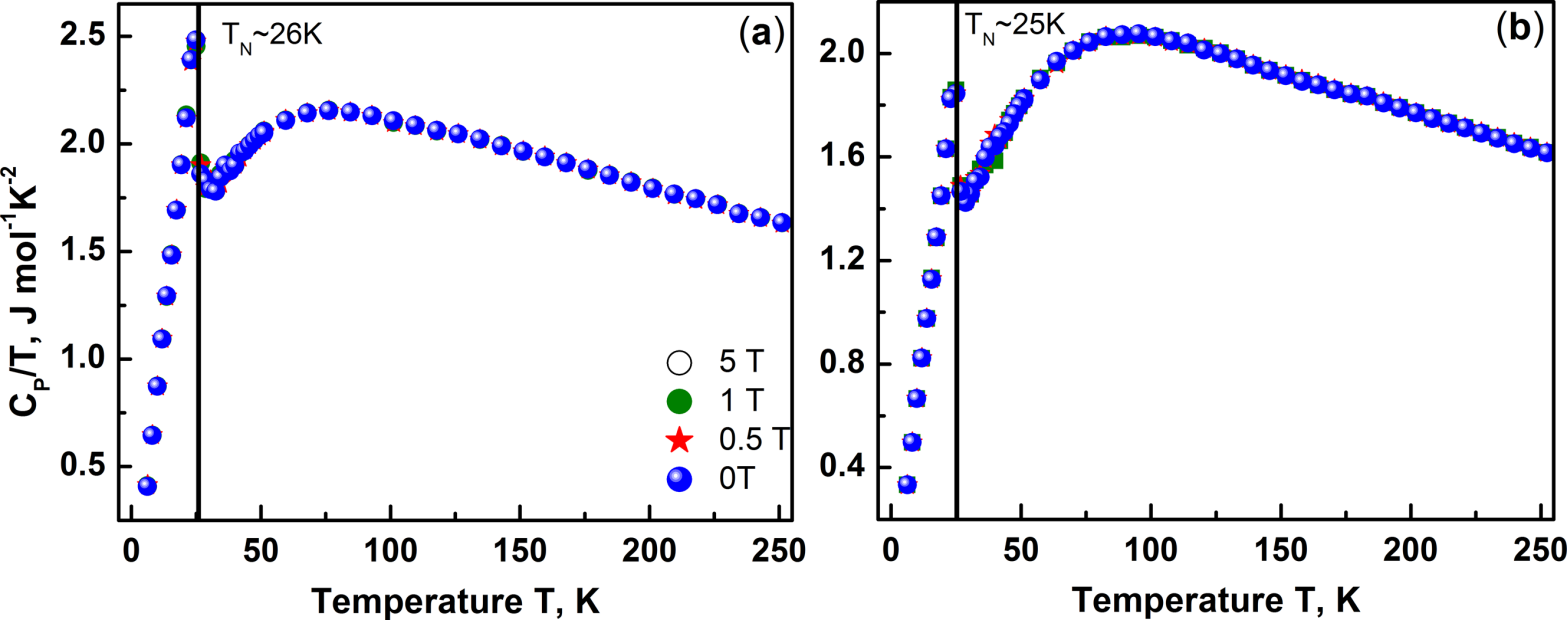
The variation of specific heat capacity C_p_ both as a function of temperature T and magnetic field H shows a clear peak at T_N_ with a broad hump in the range 40 K to 100 K in (a) BNFSO and (b) SNFSO. The hump signals the onset of magnetic short range order with the peak indicating the formation of long range antiferromagnetic entities.

The heat capacity C_P_ measured both as a function of temperature T in the range 5 K to 300 K and magnetic field H up to 5T is shown in Figure 5. The heat capacity shows a clear peak at all fields at 26 K for BNFSO and at 25 K for SNFSO corresponding to antiferromagnetic phase transition T_N_ in both the compounds. The heat capacity is found to be nearly independent of magnetic fields at all temperatures i.e. the zero field and field dependent C_p_ overlap at all temperatures. An additional feature noticed is the presence of a broad hump in the temperature range 40 K to 100 K in both the compounds and is attributed to onset of ordering with the consequent release of entropy^19^. The magnetic contribution to the heat capacity however cannot be extracted without knowing the exact non-magnetic or phonon contribution. Since the compositions studied in the present work all have Fe^3+^ this could not be done. The absence of magnetic field dependence of C_p_ in both BNFSO and SNFSO indicates that the spin configuration does not change with changing field and that the magnetic order is inherent to the system. The strong temperature dependence as well as the hump like feature in the heat capacity indicate that both correlated as well as orphan spins influence the heat capacity and the orphan spins could be due to the presence of a variety of structural defects which can result in the formation of magnetically different phases^26^,^27^.

### Magnetization (dc and ac)

**Figure 6:**
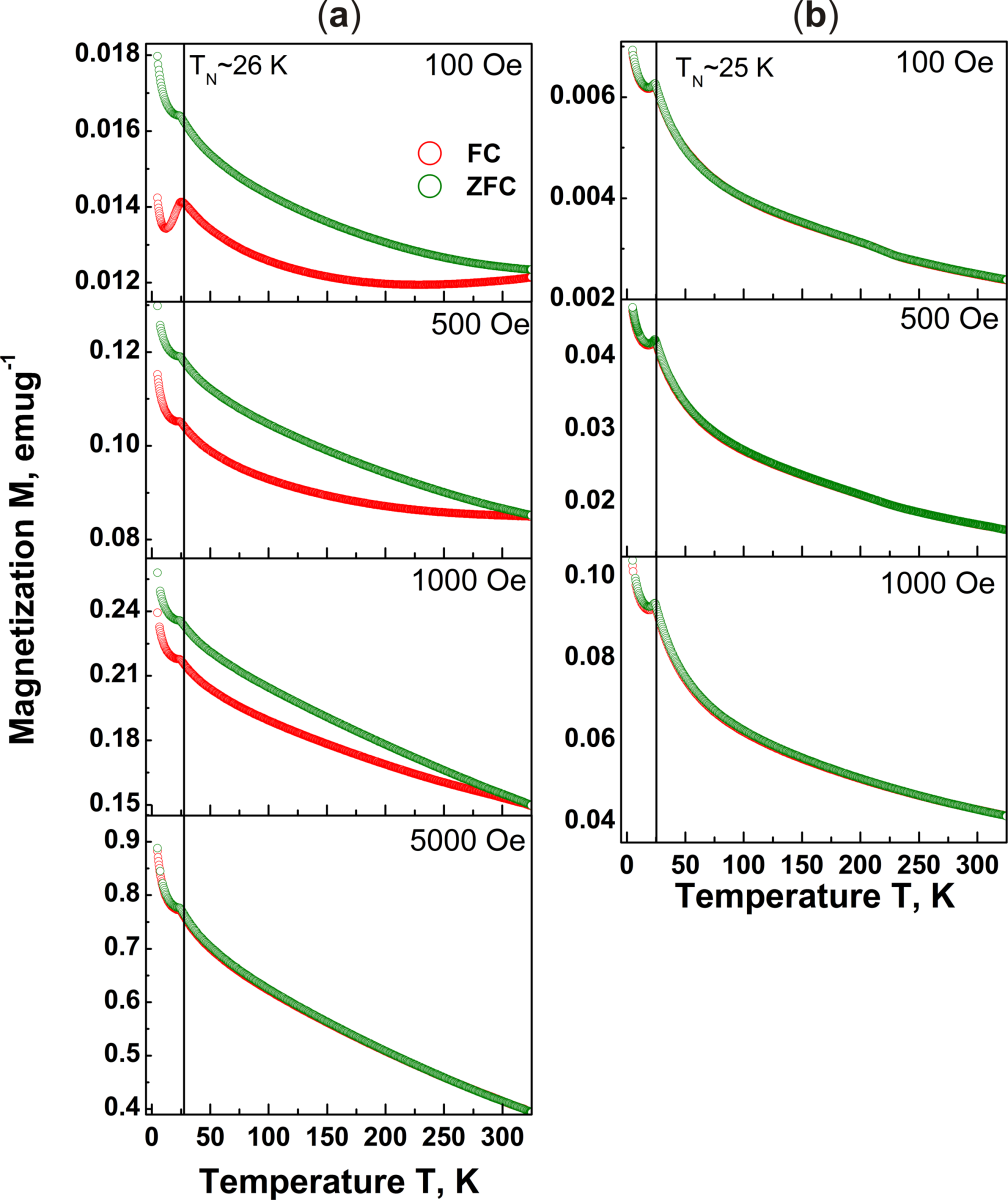
The variation of specific magnetization M with T and H shows path dependent irreversibility in BNFSO, (a). The field cooled (FC) and zero field cooled (ZFC) magnetizations diverge for T < T_irr_ and T_irr_ decreases with increasing H, a behavior typical of magnetic glasses. The transition at 26 K, T_N_, however is independent of ‘H’. In the case of SNFSO (b) however the magnetization does not exhibit any irreversibility with only a phase transition at 25 K, T_N_ at all fields.

The dc magnetization also exhibits a clear field independent cusp at 26 K and 25 K respectively for BNFSO and SNFSO corresponding to the antiferromagnetic transition as seen in Figure 6 and is in agreement with heat capacity data. The similarity between the two compounds BNFSO and SNFSO; and the earlier reported studies^28^ on BNFSO however ends here. The magnetic field and path dependence of the magnetization M, i.e. zero field cooling and field cooling, exhibits clear irreversibility in the case of BNFSO which is absent in SNFSO. The irreversibility temperature T_irr_ decreases with increasing magnetic field and becomes ‘0’ for fields >5000 Oe. These results indicate the presence of a ‘spin-glass’ like phase in BNFSO which is not observed in SNFSO. The magnetic field dependence of T_irr_ in the case of BNFSO however does not follow the de-Almeida-Thouless phase boundary relation^29^, indicating an unconventional ‘glassy’ behaviour. The high temperature magnetization in the case of BNFSO follows Curie-Weiss behaviour only for T > 200 K and an extrapolation gives a value of ~−350 K for θ_CW_ the Curie-Weiss temperature compared to ~−90 K for SNFSO. These values show that the level of geometric frustration in the case of BNFSO is much greater than that present in SNFSO, ~3.6. The atomic magnetic moment determined from the Curie constant for BNFSO is found to be ~5.73 μ_B_ compared to 5.79 μ_B_ for SNFSO. The lower than expected magnetic moment, 5.92 μ_B_, is attributed to the frustration present in the structure and the extent of covalency in the Fe^3+^ electrons. Hence, it can be concluded that the additional feature observed in the heat capacity and the irreversibility in magnetization in the case of BNFSO are solely a consequence of short range magnetic effects such as formation of ‘spin-glass’ like phase. The presence of ‘spin-glass’ phase in the case of BNFSO is further confirmed by ac magnetic susceptibility studies discussed in the next paragraph. In the case of SNFSO however the short range ordered magnetic entities do not seem to affect the dc magnetization.

**Figure 7:**
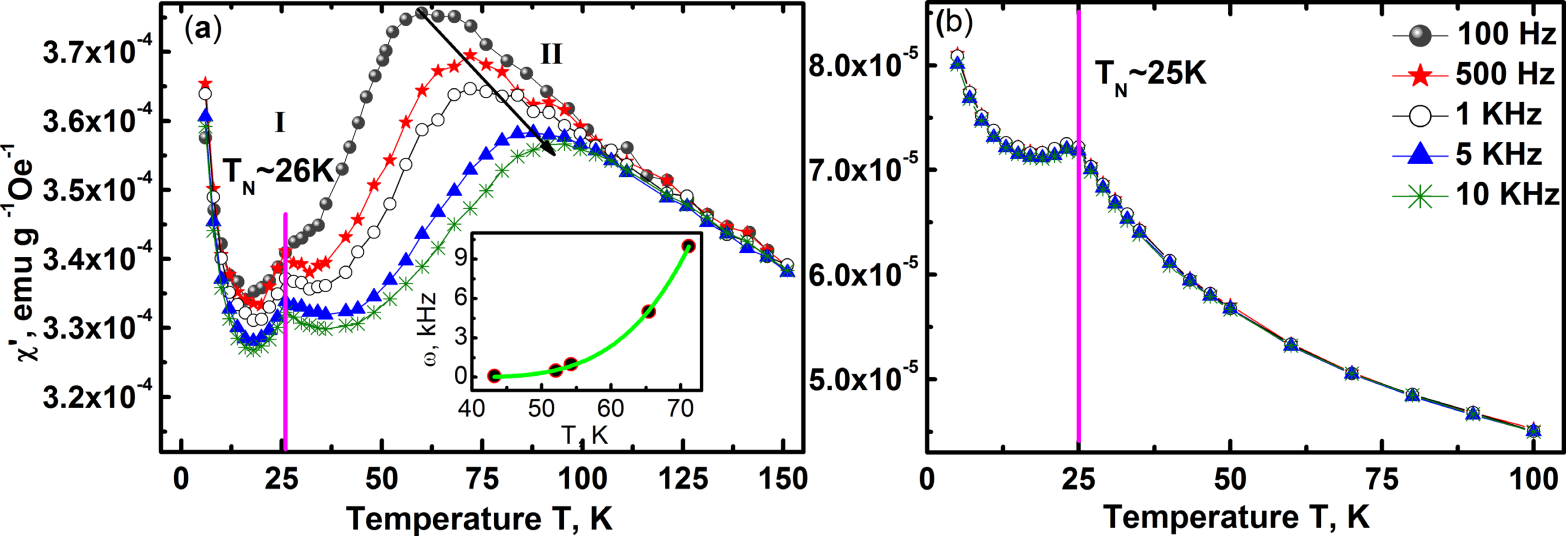
The real part of the ac magnetic susceptibility measured as a function of frequency ω and temperature T of BNFSO (a), shows a frequency independent peak at 26 K, T_N_ and a frequency dependent dispersive peak in the range 40 K to 90 K. The temperature dependence of the peak frequency fitted to the critical slowing dynamics model, power law dependence, is shown in the inset. The susceptibility of SNFSO on the other hand shows a single, non-dispersive peak at 25 K with no other peaks, (b).

The frequency ω and temperature T dependence of ac magnetic susceptibility, shown in Figure 7(a) shows two clear peaks – a ω-independent peak at 26 K and a broad dispersive peak in the temperature range 40 K to 80 K. The ω-independent peak at 26 K corresponds to the magnetic phase transition into an antiferromagnetic state and is ‘athermal’ in nature. The second peak in the range 40 K to 80 K however is highly dispersive and shifts to higher temperatures with increasing ω. The width of the peak decreases with decreasing frequency, a characteristic feature of spin glass systems. The shift of peak temperature 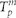 with frequency ω given by the factor 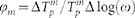 is generally used to qualitatively determine the type of magnetic behaviour^31^. In the present case of BNFSO it is found to be ~0.2, a value higher than that corresponding to a prototypical spin glass but much smaller than that for a superparamagnetic system. The magnetic freezing temperature 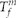 for crossover from a paramagnetic state to a glass with critical slowing spin dynamics can be obtained using the relation;
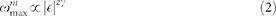
where *ε* represents the reduced temperature 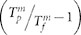 and *zγ* is the critical exponent. The magnetic freezing temperature 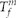 is found to be 34.7 K, indicating that the high temperature glassy clusters phase is frozen above T_N_ and this phase further transforms to the antiferromagnetic phase at 26 K. The critical exponent is found to be 3.9, corresponding to the 3D Ising model^30^ with a slowing time for collective particles relaxation of 1.2 × 10^−4^ s. These results clearly show that the dispersive magnetic susceptibility is due to the formation of a cluster glass phase. The ac magnetic susceptibility in the case of SNFSO, Figure 7(b), however is very different compared to that observed in BNFSO. The susceptibility exhibits a single frequency independent peak at 25 K, T_N_, with no other high temperature peak. This clearly shows that SNFSO undergoes just one phase transition from the paramagnetic state to antiferromagnetic state, in contrast to the behaviour observed in BNFSO where in this transition is mediated via the glassy state.

**Figure 8:**
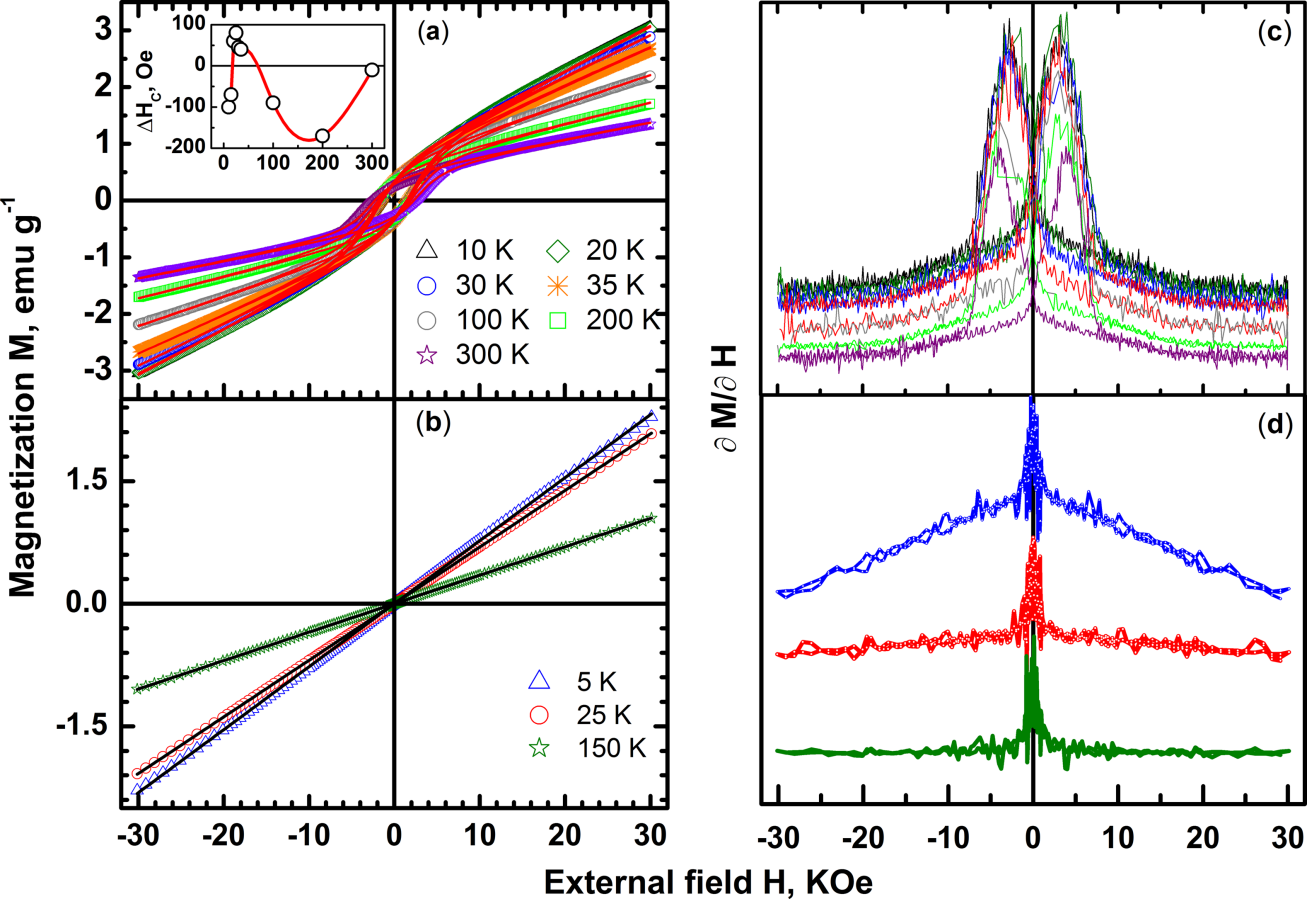
The hysteresis of specific magnetization M with field ‘H’ and a non-saturating high field behaviour at constant temperature in the case of BNFSO indicates presence of a ferromagnetic component at all temperatures along with a antiferromagnetic component (a). The specific magnetization of SNFSO however does not show any hysteresis indicating the presence of a single phase at any T and H, (b). The derivative of the specific magnetization dM/dH as a function of H clearly illustrates the hysteretic nature, (c) and (d). The coercivity H_c_ also exhibits exchange bias, ΔH which changes sign below T_N_ and above T_N_ showing the presence of an antiferromagnetic component together with the ferromagnetic component in BNFSO and is shown in the inset of (a).

The isothermal variation of magnetization M with magnetic field H at temperatures both below and above T_N_ has also been investigated and the results are shown in Figure 8 for both BNFSO and SNFSO. The magnetization does not saturate in both the cases for fields as large as 3 T, clearly showing the paramagnetic/antiferromagnetic nature above and below T_N_ respectively. The magnetization in the case of BNFSO is clearly due to at least two distinct magnetic phases at any temperature, below and above T_N_. This can be seen clearly in the derivative plot shown in Figure 8(b). The derivative of magnetization 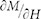 is highly non-linear with peaks at low fields and tends to have a constant value at large fields – behavior typical to a mixture of ferromagnetic and paramagnetic/antiferromagnetic phases. The magnetization exhibits a clear coercive field H_c_ which increases from 1700 Oe to 2500 Oe with increasing temperature in the entire temperature ranging from 5 K to 300 K. In systems wherein ferromagnetic and antiferromagnetic phases coexist, exchange bias phenomenon has been extensively observed. In such systems the isothermal hysteresis loop shifts its center away from the origin and exhibits asymmetry. The extent of this shift is defined as the exchange bias field H_E_ and this field is a measure of the strength of the coupling between the two phases. It is to be noted that the hysteresis behavior in the present work also exhibits considerable exchange bias with a peak in the bias field at T_N_ which reverses sign both below and above T_N_ clearly showing the presence of antiferromagnetic phase together with a ferromagnetic phase, Figure 8(a) inset. The low temperature biasing field sign reversal takes place between 10 K and 20 K while that above T_N_ is around 50 K and vanishes at room temperature. The reversal of sign of the biasing field with temperature clearly indicates the complex nature of the magnetic structure as well as the magnetic transitions in the case of BNFSO. The low T crossover is plausibly due to the formation of a long range ordered antiferromagnetic phase while that at high temperatures is due to formation of spin glass clusters. The dc magnetization and ac susceptibility show that multiple phases, antiferromagnetic, ferromagnetic, spin glass and paramagnetic, coexist in BNFSO in different temperature ranges. The substitution of Ba^2+^ with an isovalent Sr^2+^ changes the magnetic response completely and does not exhibit any anomalous behavior as seen in Figure 8(c). The magnetization does not exhibit any hysteresis indicative of the presence of a single phase, paramagnetic above T_N_ and antiferromagnetic below T_N_. It should however be noted that below T_N_ the system has a non-linear magnetic field response which changes to a near linear response above T_N_ typical of an ideal paramagnet, Figure 8(d). The high temperature magnetization is linear at all temperatures with the susceptibility obeying the Curie-Weiss behavior down to 100 K with a magnetic moment of ~5.8 μ_B_ in complete agreement with the magnetic moment determined in the case of BNFSO.

### Dielectric constant

**Figure 9:**
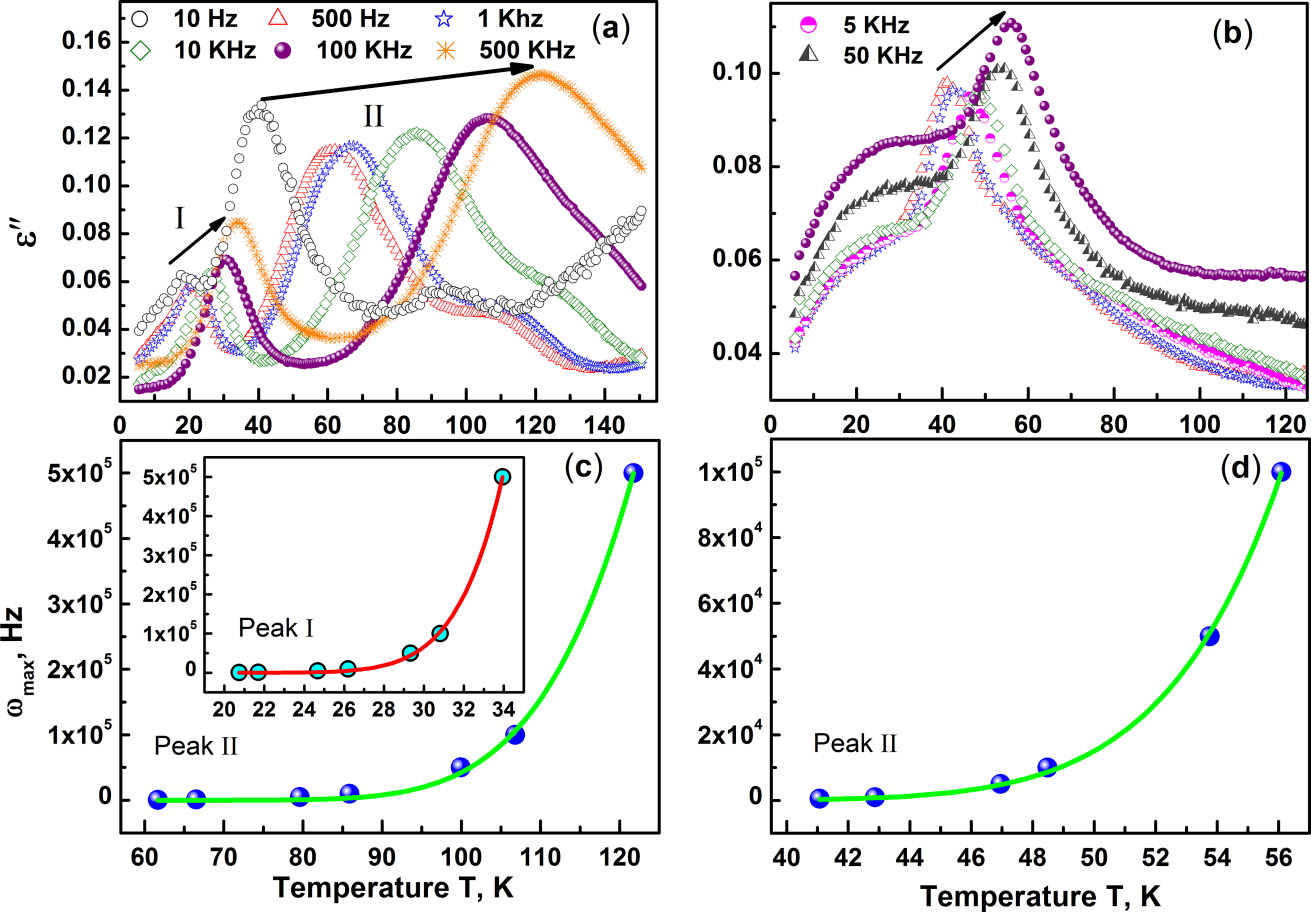
The imaginary component of the dielectric constant ε” measured as a function of frequency ω in the range 10^1^ to 10^5^ Hz from room temperature down to 5 K is shown in (a) and (b) for BNFSO and SNFSO respectively. In the case of BNFSO, *ε*” clearly shows two dispersive peaks, peak-I and peak –II in the temperature ranges 20 K to 35 K and 40 K to 125 K respectively, (a). There is only one high temperature dispersive peak in SNFSO, (b). In both the cases however ω^ε^_max_ has a Vogel Fulcher temperature dependence as seen in the insets.

The dielectric constant ε studied both as a function of frequency ω in the range 10^1^ Hz to 10^5^ Hz and temperature T for both BNFSO and SNFSO is shown in Figure 9. The dielectric constant shows distinctly different behavior between the two compounds. In the case of BNFSO two clear dispersive peaks are observed in the temperature range 20 K to 35 K (peak I) and 40 K to 125 K (peak II), Figure 9(a) while SNFSO exhibits a single high temperature dispersive peak in the range 40 K to 80 K, Figure 9(b) with a frequency independent non-dispersive hump at T_N_. The dispersive dielectric response observed in both the cases is similar to that observed in relaxor ferroelectrics wherein polar nano regions (PNR) have been known to form due to displacement of the ions from their equilibrium positions. The PNRs are known to be extremely stable and robust with extremely large fields being required to destroy them^31^. The frequency dependence of the peak temperature 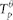 defined by the parameter 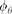, similar to that used to define the magnetic susceptibility dispersion is found to be ~0.15 for peak I and ~0.2 for peak II in the case of BNFSO. These parameters are similar to that determined from ac susceptibility studies and clearly illustrate the magnetic and electric similarities and hence a direct coupling between the two order phenomenon. The temperature dependence of frequency maximum for both the peaks and in both the compounds follows a Vogel-Fulcher type relaxation given by;
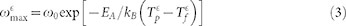
where ω_0_ is the attempt frequency, E_A_ the barrier activation energy and 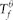 the glass freezing temperature. The activation barrier at low temperature is found to be 40 meV while that for high temperature is ~71 meV in the case of BNFSO. The activation energy in the case of SNFSO is ~65 meV, similar to that determined for BNFSO peak II. The activation energy determined for peak I in the case of BNFSO is similar to that observed in the case of Pr_0.7_Ca_0.3_MnO_3_ wherein the polarization is phonon assisted and results in the formation of polaronic charge carriers at low temperatures^32^. The low temperature freezing temperature for BNFSO is found to be 1.8 K, in agreement with low temperature magnetic structural studies which show the presence of short range clusters till ~1.5 K^33^. The freezing temperature 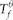 for the high temperature peak in BNFSO however is found to be 25 K, close to T_N_, while in the case of SNFSO this temperature reduces to 3.8 K indicating that the polar glass phase is present even below T_N_. This difference in freezing temperatures is due to the intervening phase transition into a second relaxor state at 26 K in the case of BNFSO. The formation of polar nano regions in these relaxors is attributed to fluctuating dipoles with the nano regions having lower symmetry compared to the surrounding matrix in which they are embedded^34^. The scale of these domains however is very small and is predicted to increase with decreasing temperature, thus making it extremely difficult to observe them experimentally.

## Discussion

Materials with frustrated antiferromagnetic interactions coupled with commensurate/incommensurate helical spiral structure are unique and exhibit a variety of unusual magnetic structures. The existence of energetically favorable multiple ground states in such materials leads to co-existence of multiple phases ranging from ferromagnetic to antiferromagnetic and various types of glassy states. The phenomenon of co-existing multiple phases is often driven by kinetic constraints which lead to arresting of phase transformations which in turn results in the formation of non-ergodic structures wherein ordered and disordered phase in-homogeneities can coexist and can be at the nanoscale or at longer mesoscopic length scales^35^. The magneto-electric coupling, if it is possible in such materials can exhibit unique features including giant higher order coupling constants which makes them extremely promising for multiferroic applications^36^. The ideal ground state in the case of BNFSO is a frustrated in-pane trimer with an incommensurate helical magnetic structure along the c-direction. This structure has been predicted to be uniquely stabilized by intra- and inter-plane super exchange and anti-symmetric DM interactions. Any changes to this delicate balance of interactions leads to the possibility of multiple magneto-electric phase’s formation with a complex behavior. The various types of defects that can change the magnetic structure and hence alter the exchange interactions are;

1. Anti-site defects such as exchange of cations belonging to different sites;
2. Truncation of the helical spin order leading to the formation of weak ferromagnetic entities; and
3. Non-magnetic grain boundary phases.

The anti-site defects can change the nature of super exchange interactions by altering the length and angle of the exchange path from antiferromagnetic to ferromagnetic as per the Goodenough-Kanamori-Anderson rule^37^,^38^,^39^. In the case of BNFSO Fe^3+^ should occupy the tetrahedral sites in the ordered crystal. However, it is known that Fe^3+^ can also occupy octahedral sites and in such cases it will lead to formation of anti-site defects – Fe^3+^ replacing Nb^5+^. This leads to changing of the super exchange path length as well as the angle resulting in variation of both antiferromagnetic and ferromagnetic interactions. Recent studies have indeed shown that Fe^3+^ occupies two non-equivalent sites and this can lead to a structural transition resulting in the formation of either a monoclinic phase or just result in reducing the symmetry to stay in the trigonal class with P3 symmetry^25^,^40^. The antiferromagnetic structure in BNFSO is stabilized by the strong in-plane exchange interaction parameter J_1_ and the out-of-plane parameter J_5_. The exchange length corresponding to these two exchange parameters in both BNFSO and SNFSO has been determined from structural refinement and is given in Table 1. The in-plane exchange length corresponding to J_1_ in both the cases has been found to be identical while the out-of-plane exchange length corresponding to J_5_ in SNFSO is longer compared to that in BNFSO. This is because the ionic radius of Sr^2+^ is less than that of Ba^2+^ which results in stretching and possibly bending of the bonds. These results indicate that the magnetic defect structure in the two cases is different. However the absence of macroscopic structural changes in the present case in the complete temperature range down to 6 K indicates that anti-site defects formation is a local phenomenon with extremely short length scales and leads to formation of electronically phase separated multiple phases. Interestingly however, substitution of Ba^2+^ with Sr^2+^ changes the nature of electronic phases predominance. Only one type of phase both in terms of magnetic and electric behavior, is present at any given T and H. Substitution appears to lead to the formation of ordered structure with no intermediate magnetic transitions. Electrically however the Sr^2+^ substituted system still shows a relaxor like behavior before it transforms into a long range ordered ferroelectric phase at T_N_.

The role of structural manifestations, conventionally categorized as defects, on the electro-magnetic properties of materials in general is well known. Specifically, in the case of perovskite structures these phenomena have been extensively investigated. Substitution of A-site cation in the antiferromagnetic compound AMnO_3_ has been found to increase ferroelectric instability with increasing lattice parameter. Incorporation of Ca through Sr to Ba at the A-site leads to this increased lattice parameter and hence instability as the Mn^4+^-O^2−^Mn^4+^ superexchange bond angle changes. In the case of Sr_0.5_Ba_0.5_MnO_3_, ferroelectricity was observed together with antiferromagnetic ordering due to off-centering of Mn^4+^ ions inspite of having a cubic structure^41^. The ferroelectricity however was reduced due to the presence of small length scale defects such as twinned tetragonal domains. In the case of n = 2 Ruddelsden-Popper compound Ca_3_Mn_2_O_7_, polarization was predicted due to rotations of the two nonpolar lattice modes which also induced weak ferromagnetism together with magnetoelectricity^42^. This phenomenon has indeed been found to be present in the heterostructures made of nonpolar YFeO_3_ and LaFeO_3_^43^. The polarization and magnetoelectric coupling in these heterostructures is due to a combination of tilting of (BO_6_)^9−^ octahedral and ordering of A and B-site cations, both being nanoscale structural effects. It was also found that only ~25% of the heterostructure was polar while the other regions were nonpolar due to the formation of twin defects. A similar effect has been observed recently in the case of the layered perovskite (Ca_y_Sr_1-y_)_1.15_Tb_1.85_Fe_2_O_7_ wherein octahedral tilting was a result of underbonding caused by reduction of A-site cation size. This resulted in the exhibition of polarization and magnetization above room temperature in this compound^44^. These recent results clearly show that extremely short length scale structural ‘defects’ can indeed change the macroscopic physical properties such as observed in BNFSO and SNFSO.

**Figure 10:**
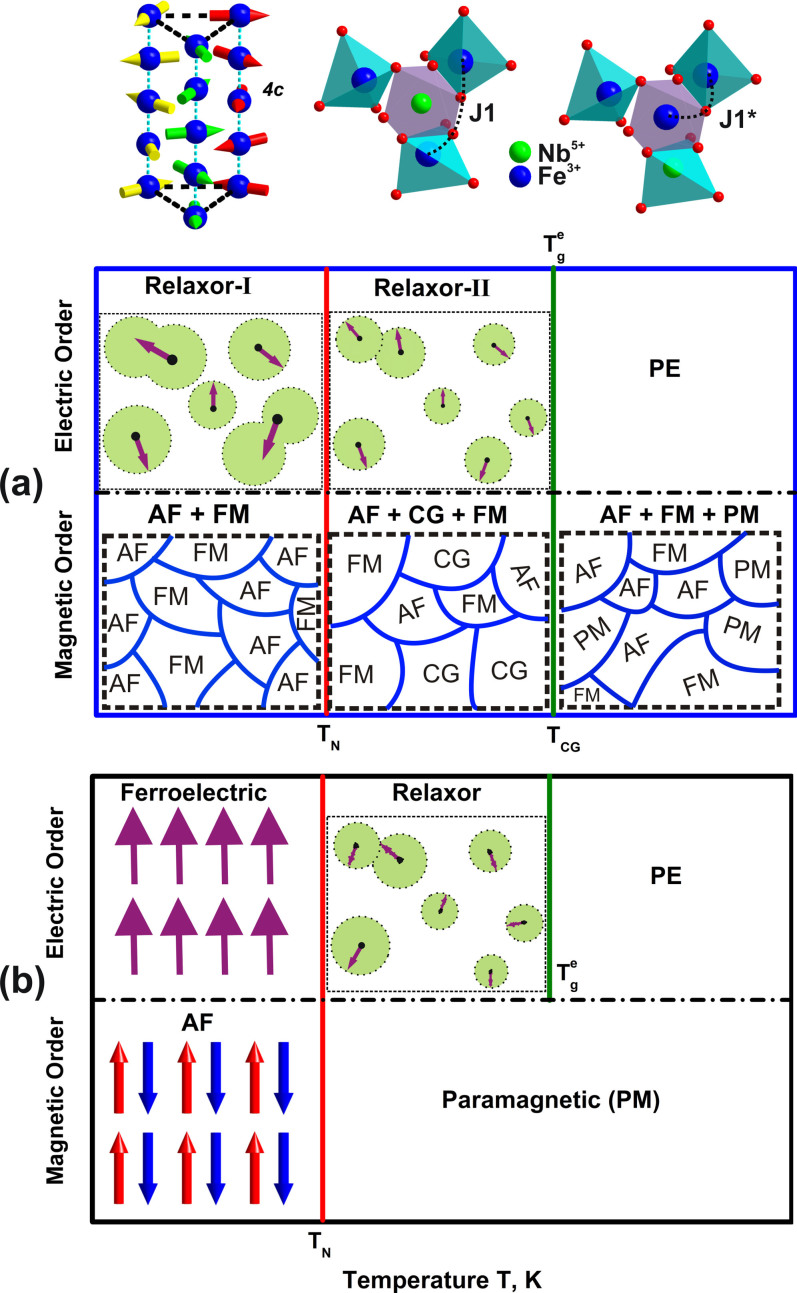
A phenomenological representation of magnetic and electric structures present at different temperatures in BNFSO (a) and SNFSO (b). The incomplete magnetic helical structure and modification of the in-plane super-exchange interaction J_1_ due to an antisite defect are shown schematically on the top. AF, FM, CG, PM and PE represent antiferromagnetic, ferromagnetic, cluster glass, paramagnetic and paraelectric phases.

The above described magnetic and electric behaviors of BNFSO and SNFSO can be summarized by a phenomenological phase stability predominance diagram as shown in Figure 10. Magnetically, BNFSO exists in multiple phase separated states at any temperature in the range 5 K to 300 K whose length scales are extremely small. For T > 100 K, paramagnetic, ferromagnetic and antiferromagnetic phases exist in the system and the isothermal magnetic hysteresis combined with ac magnetic susceptibility studies point to the existence of these phases. On cooling to 40 K < T < 100 K, the composition of the magnetic phases changes. A cluster glass phase is present together with ferromagnetic and antiferromagnetic phases in this temperature range. On further cooling to T < 40 K the system transforms to a mixture of antiferromagnetic and ferromagnetic phases. The antiferromagnetic phase present at all temperatures provides the exchange bias to the ferromagnetic phase. The presence of multiple phases is plausibly due to entities such as anti-site defects and termination of the helical magnetic structure into a partial helix as shown on the top in Figure 10. In comparison to the complex magnetic phases distribution in BNFSO, the scenario in the case of SNFSO is simple and ‘clean’. The system exists either in the paramagnetic state above T_N_ or antiferromagnetic state below T_N_. A complete absence of co-existence of multiple phases clearly shows that a smaller Sr^2+^ ion in the same dodecahedral site results in significant magnetic structural stabilization. The dielectric studies on the other hand for the two compounds are relatively similar. In the case of BNFSO a true ferroelectric phase transition is not clearly observed. The system transforms to a low temperature relaxor phase from the high temperature paraelectric phase via a second relaxor phase. These results are in complete agreement with the magnetic results which exhibit complex phenomena. The Sr-substituted compound on the other hand exhibits a relatively simple transition into the ferroelectric state at low temperatures mediated by the relaxor phase. In both the compounds the dielectric studies reflect the magnetic results. The similarity in either the complexity observed in BNFSO or the simplicity observed in SNFSO clearly illustrate that the magnetic and electric orders in the two compounds are strongly correlated.

## Conclusions

The magnetic and dielectric properties of Fe-containing langasite compounds Ba_3_NbFe_3_Si_2_O_14_ and Sr_3_NbFe_3_Si_2_O_14_ have been investigated in detail using a combination of structural and physical properties characterization down to 5 K. The main difference between the two compounds is the alkaline earth metal cation – Sr^2+^ and Ba^2+^, which are isovalent but with different size. The two compounds however exhibit completely different magnetic and dielectric properties. The Ba-compound has multiple co-existing magnetic phases including a glassy phase due to electronic phase separation at extremely small length scales which are not detected by structural studies. All the different phases however exhibit their distinct nature which is revealed either in dc or ac magnetization studies as well as dielectric studies. A glassy behavior in the temperature range 40 K to 90 K is observed both in ac susceptibility results as well as in dc magnetization studies which show a decreasing thermal hysteresis with increasing magnetic field. The Sr-compound on the other hand is relatively simple without any coexisting magnetic phases. Both the compounds however exhibit a relaxor like glassy dielectric behavior above 40 K before the onset of antiferromagnetic transition. The presence of magnetic and dielectric glassy phases in the Ba-compound in the overlapping temperature ranges clearly illustrates the strong coupling between the two orders. Substitution of isovalent Sr in place of Ba however prevents the formation of a glassy magnetic phase. The formation of a relaxor like phase before the onset of antiferromagnetic and ferroelectric transition also shows the strong correlation between the two orders but of a different nature. The existence of disordered glassy phases indicates that these compounds can have large higher order magneto-electric coupling constants, β_ijk_ and γ_ijk_ which are not governed by structural symmetry constraints.

The difference in behavior between the two compounds, Ba- and Sr-, is plausibly due to differences in super-exchange path length and angle which can stabilize either the antiferromagnetic or ferromagnetic interactions. Also, in the case of Ba_3_NbFe_3_Si_2_O_14_ the presence of anti-site defects and incomplete magnetic helical arrangement have been found to result in the formation of multiple magnetic phases. These results clearly illustrate the role of frustration – geometric and magnetic, on the presence of multiple ground states which lead to complex phase separation phenomena. The present study shows that ‘frustrated’ systems in general exhibit complex and varying behaviors depending on a variety of factors. The presence of various types of small scale structural defects is found to change dramatically the behavior exhibited by relatively defect free single crystals. A complete understanding and predictive capability of the behavior of such materials is far from complete and needs more exhaustive theoretical studies to complement the experimental results.

## Author Contributions

S.V. conceived the work while S.S.R. carried out all the experimental work including synthesis of samples. S.V. and S.S.R. analyzed the data and S.V. wrote the manuscript. Both the authors discussed all results and the manuscript.

## Additional Information

**How to cite this article**: Rathore, S.S. & Effect of divalent Ba cation substitution with Sr on coupled ‘multiglass’ state in the magnetoelectric multiferroic compound Ba_3_NbFe_3_Si_2_O_14_ *Sci. Rep.* **5**, 9751; doi: 10.1038/srep09751 (2015).

## Figures and Tables

**Figure 1:**
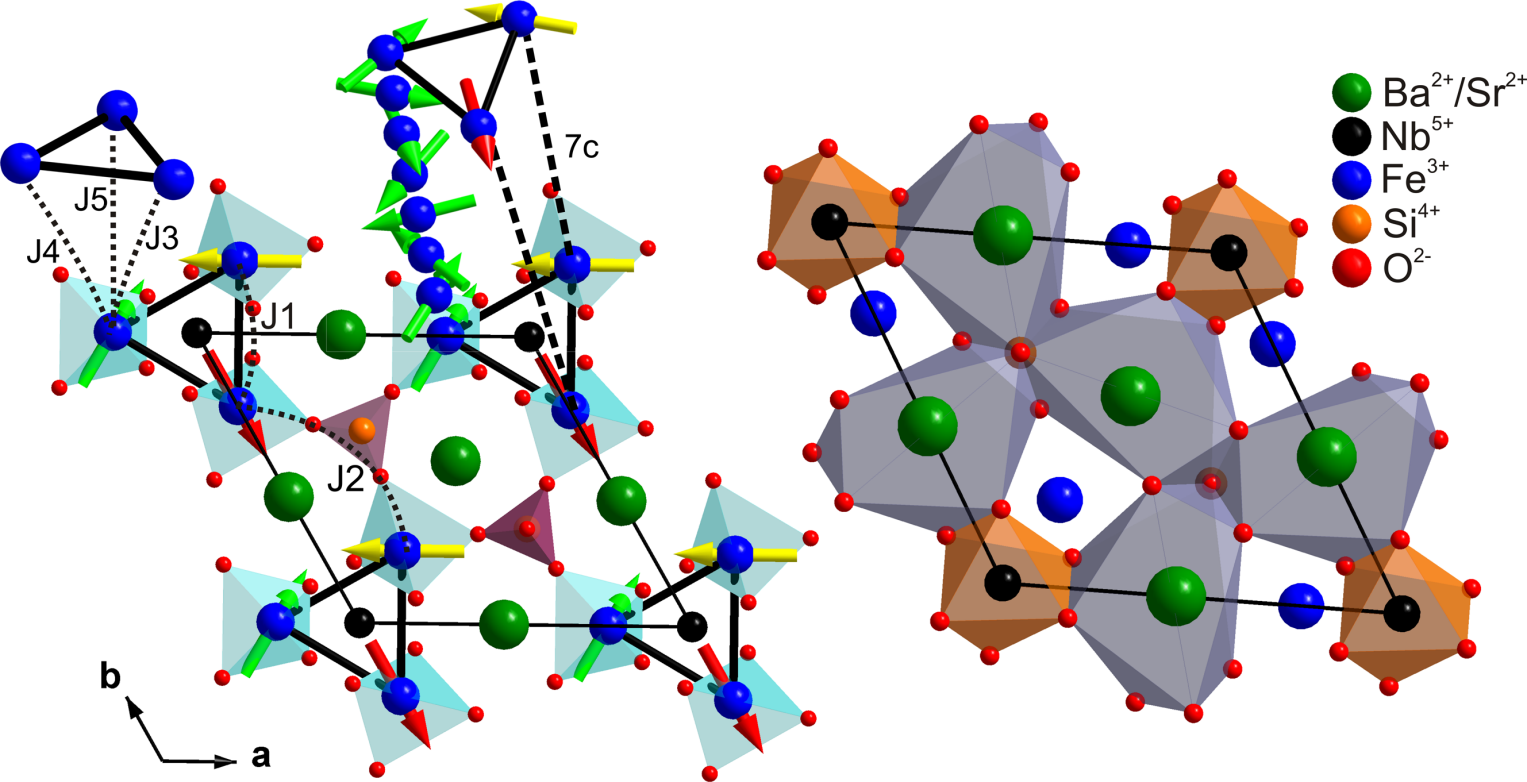
Schematic diagram showing the arrangement of various cations in the Fe-substituted langasite compound (Ba/Sr)_3_NbFe_3_Si_2_O_14_. The ab-plane magnetic interactions J_1_ and J_2_ and the intra-plane interactions J_3_, J_4_ and J_5_ which result in the formation of trimer and helical spin structures are shown. The incommensurate structure along the c-direction has a length of 7c.

**Table 1:** The crystallographic parameters obtained from refinement of room temperature diffraction pattern for Ba_3_NbFe_3_Si_2_O_14_ (R_Bragg_ = 2.0; R_WP_ = 2.6; χ^2^ = 1.4) and Sr_3_NbFe_3_Si_2_O_14_ (R_Bragg_ = 15; R_WP_ = 20; χ^2^ = 2.88) respectively. The exchange path for strongest magnetic interaction J_1_ (*, in plane) and J_5_ (+, out of plane) are also given at the bottom of the table. The structural parameters for Sr_3_NbFe_3_Si_2_O_14_ are shown in parentheses, [ ]

Atom	Wyckoff	x	y	z	Occup.
Ba (Sr)	3e	0.4347(6) [0.4340(3)]	0	0	0.5
Nb	1a	0	0	0	0.166
Fe	3f	0.7525(3) [0.7531(5)]	0	0.5	0.5
Si	2d	0.333	0.666	0.4828(1) [0.5170(4)]	0.333
O1	2d	0.333	0.666	0.7769(2) [0.850(6)]	0.333
O2	6g	0.4728(5) [0.498(2)]	0.2984(5) [0.8238(6))]	0.6471(8) [0.675(3)]	1.00
O3	6g	0.2142(5) [0.2609(6)]	0.0997(5) [0.0916(7)]	0.2233(6) [0.739(3)]	1.00
* Fe-O3-O3-Fe, Å		**+ Fe-O3-O3-Fe**, Å		**a**, Å	**c**, Å
6.43 (2)		6.447(5)		8.5001(1)	5.2267(1)
[6.42(3)]		[6.621(3)]		[8.2605(2)]	[5.1298(1)]
